# Setting Requirements for a Dashboard to Inform Portuguese Decision-Makers About Environment Health in an Urban Setting

**DOI:** 10.3389/fpubh.2022.837433

**Published:** 2022-06-10

**Authors:** Marta Salgado, Paulo Nogueira, Anália Torres, Mónica D. Oliveira

**Affiliations:** ^1^Centre for Management Studies of Instituto Superior Técnico (CEG-IST), Instituto Superior Técnico, Universidade de Lisboa, Lisboa, Portugal; ^2^Institute of Environmental Health (ISAMB), Faculdade de Medicina, Universidade de Lisboa, Lisboa, Portugal; ^3^Área Disciplinar Autónoma de Bioestatística (Laboratório de Biomatemática), Instituto de Saúde Preventiva e Saúde Pública, Faculdade de Medicina, Universidade de Lisboa, Lisboa, Portugal; ^4^Valorsul, Estação de Mercadorias Bobadela, Plataforma Ribeirinha CP Lisboa, Lisboa, Portugal; ^5^IBB-Institute for Bioengineering and Biosciences, Instituto Superior Técnico, Universidade de Lisboa, Lisboa, Portugal

**Keywords:** urban environmental health, dashboard requirements, design-cards, user-centered approach, decision making

## Abstract

Dashboards are being increasingly used in the health field, and literature points out that accurate and efficient dashboards require not only dealing with data issues, but also ensuring that dashboards are user-friendly and that incorporate users' views and needs. The integration of evidence and data into decision aiding tools, such as dashboards, to assess and monitor environmental health (EH) in urban settings requires careful design. Departing from EH evidence and making use of the views of EH stakeholders and experts, this study aimed at defining requirements for a dashboard to help decision-makers analyzing and visualizing EH information in the Lisbon urban context. In order to set those requirements, it was combined a user-centered with a design card approach to engage EH potential end-users so as to collect their visualization preferences and gather information related to dashboard requirements. Specifically, three online group semi-structured interviews, involving 11 potential end-users from different organizations, were conducted; design cards with a set of visualization options regarding 17 indicators of built and natural environment determinants were used in the interviews to capture participants' preferences and their rationale; questions about other dashboard features were also asked; and the results from the interviews were synthesized into four separate, but interrelated features, and operationalized into 11 requirements for a dashboard to monitor EH in Lisbon. This study contributes to EH literature by producing knowledge to inform dashboard construction, by highlighting issues related with the usability, analysis, and visualization of data to inform EH decision-making in urban contexts, and by designing an approach that can be replicated to other EH dashboard contexts.

## Introduction

New digital tools are emerging rapidly, with health and environmental institutions increasingly collecting larger amounts of data and facing novel opportunities to adopt technologies to manage such data for improved decision-making (1–3). Nevertheless, the way data is being gathered, analyzed and reported to policymakers has been far from enabling structured analyses and from identifying relevant policy issues in the environmental health (EH) domain (4).

Common challenges related with health data have been data standardization; database linkage, integration and sharing; selection of key data for decision-making; and finding a balance between evidence and research data to be included within decision tools (5, 6). Challenges related to translating data into better decision-making concern the creation of decision aiding tools in which evidence and data are understandable and inform about the extent to which specific policy goals are attained (7, 8), and tailoring such tools to the specific settings (9). This incorporation of data within decision aiding tools requires engaging organizations and policymakers in the early stages of design of the tools (10), which can then contribute to a shared awareness about data concerns and also to motivate organizations to improve data consistency and accuracy (11, 12). Nevertheless, it is not common to make use of structured formats to formally engage organizations and policymakers in the process of building decision aiding tools.

Multiple health and EH institutions have been investing in tools using visual formats to help communicating information to EH stakeholders and enhance decision-making. For instance, the European Environment Agency has developed a visualization tool in which it is possible to track and compare national data (collected by European countries) concerning air quality, noise, mobility, and housing conditions indicators (13). This type of tool is powerful to monitor EH and enables analyses and reflection by national and multinational authorities (14).

Among such decision aiding tools using visual formats, dashboards have increasingly being adopted (15), with its usefulness being widely recognized in the health field. Health dashboards portray information from various databases while making a visual display of the indicators whose performance has been shown to impact health outcomes (15, 16). The development of health dashboards has been attracting interest both from academia and industry which develop novel dashboards and collect data, and from the government which needs to monitor the evolution of health indicators and to foster evidence-based policymaking (17). Following trends observed in other healthcare areas, EH institutions have been trying to implement digital tools to manage data and monitor performance of indicators, including dashboards (18). Although the monitorisation of EH in urban settings has been considered essential to improve health and EH interventions (19, 20), there are few tools to monitor EH in that context (14) and there has been no consensus on how to develop such tools (20, 21); and it is known the complexity entailed in analyzing EH at the local level, which partly explains the lack of tools to monitor EH (21). This complexity is partly related with the need to make available a wide range of built and natural environment indicators' data to address their multifactorial effects on health, as well as with data quality and completeness issues (22).

Designing a dashboard is far from being straightforward (23), and meeting end-users' requirements has been shown to be a key factor for achieving users' expectations, preferences and needs, as well as to build sustainable tools (24, 25). Shah and Robinson (26) stated that understanding users' requirements during design determines the success or failure of a tool. To enable the building of decision aiding tools with the potential to reflect users' views, approaches like user-centered design and design card methodologies have been used in the early stages of dashboard's construction. The user-centered approach engages end-users so as to gather their analytical needs and to select data, information, and visualization preferences to meet their goals (27). This approach was implemented to develop urban dashboards to monitor indicators such as quality of life and urban sustainability for cities like London, Dublin, and Chicago (28, 29). The design card methodology has been used to facilitate a shared understanding and communication among designers and end-users, helping to kick off and to guide and structure the discussion (30). The combination of user-centered design with design card has already been used in the contexts of developing game-based learning practices (31) and cardiovascular devices for older adults (32), and can be adapted and enhanced to other contexts.

Lisbon has been recently nominated as European Green Capital 2020 and promoting several EH-related initiatives, as well as expressing the need and scope to develop and implement tools like dashboards to evaluate EH (33). There being a lack of tools to monitor EH at the local and urban context (5), there being a need to develop a dashboard to monitor EH in Lisbon, as well as scope for a proper elicitation of dashboard user requirements to inform the construction of EH monitorisation tools in urban settings (1), this study aims at developing and implementing methods to identify users' requirements and expectations to feed the construction of a dashboard to monitor EH in the Lisbon city. To that end, this study combines a user-centered approach with design cards to engage potential EH Lisbon end-users through online group interviews; and information gathered in interviews is synthesized into a set of requirements to build a dashboard. This study contributes to EH literature by producing knowledge and insights to inform dashboard construction, by highlighting issues related with dashboard usability, analysis, and visualization of data to inform EH decision-making, and by designing an approach to engage end-users that can be replicated to other EH dashboard contexts.

## Materials and Methods

**Figure 1:**
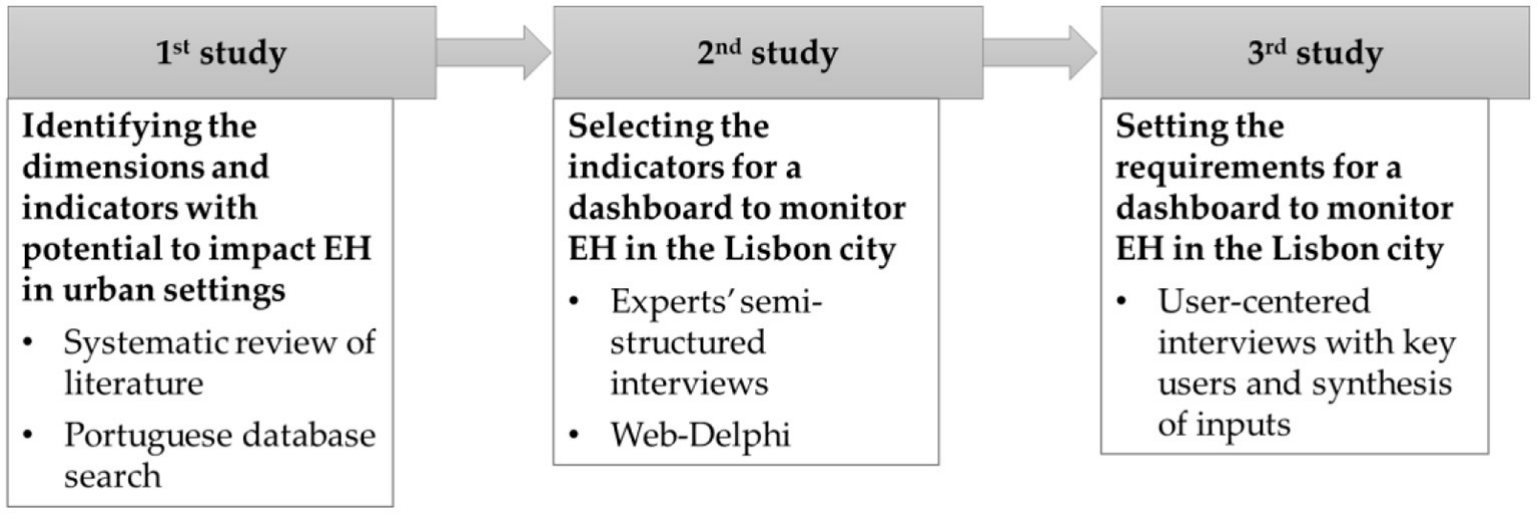
Overview of the research process adopted to move toward the design and construction of an evidence-based dashboard to monitor EH in the Lisbon city.

Aiming to identify the requirements for a dashboard to monitor EH in urban settings, taking Lisbon as a case study, this work succeeds two previous studies: in a first study the evidence on which indicators are relevant for analyzing EH in urban settings was gathered through a systematic review of literature described in detail in Salgado et al. (34); and in a second study a participatory approach was implemented with Portuguese EH stakeholders and experts to select a comprehensive set of built and natural environment indicators suitable to assess and monitor EH in the Lisbon urban context (5). This study aims to further advance in the involvement of EH stakeholders and experts in the definition of requirements for a dashboard to support EH monitoring and decision-making in the Lisbon urban context. Accordingly, results from the two previous studies are an input to this study (Figure 1). Potential end-users participate in setting the requirements for the dashboard by consulting information and data relevant to the analysis and monitoring of EH in Lisbon. Participants involved in this study have not participated in the previous studies.

### Selected Indicators to Monitor EH in Urban Settings

A systematic review of the literature reported in Salgado et al. (34) identified built and natural environment dimensions and indicators for which there was evidence of impact on health outcomes in urban settings. It resulted in a summary of 34 indicators grouped into nine EH dimensions. An additional search of data complemented this search to identify these indicators in Portuguese national and local databases that could be used to analyse EH in Lisbon.

**Figure 2:**
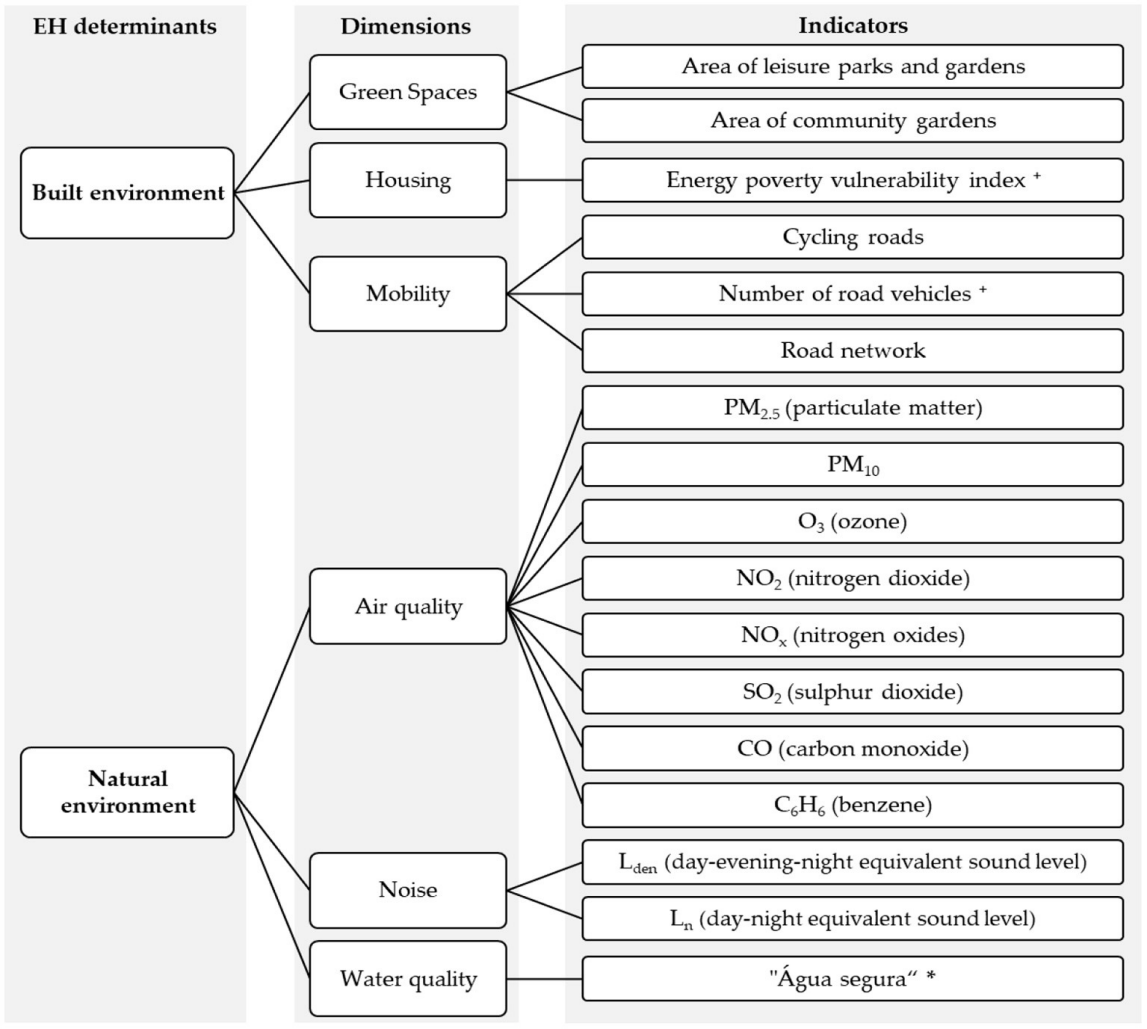
Indicators selected and validated as relevant to monitor EH in Lisbon city in Salgado et al. (5), organized by EH dimensions and determinants. ^**+**^ indicators without collected data in Lisbon. * Portuguese indicator that measures the percentage of compliance with the parametric values established in the legislation.

Drawing on the evidence and data collected, a wide number of Portuguese EH experts were involved in selecting and validating built and natural environment indicators relevant to monitor EH in Lisbon [details in (5)]. A mixed-methodology was implemented combining a set of 12 semi-structured interviews and a two-round Web-Delphi process, to validate a list of 17 relevant indicators deemed as relevant for monitoring monitor EH in the Lisbon urban setting (Figure 2).

### Designed Approach for Setting Users' Requirements

This study encompassed a series of semi-structured interviews with potential end-users from different local organizations. A user-centered approach was used to facilitate a ground-up conceptualization of data requirements from the user standpoint (35, 36). The semi-structured interviews focused on assessing the design of existing dashboards; on choosing the best visualization options for natural and built environment indicators; and on discussing user needs and additional features for a dashboard to monitor EH in Lisbon city.

### Participants

Key local government institutions engaged in environmental and health regulation, policymaking, and urban sustainability were firstly identified. Then a multidisciplinary group of potential end-users—with knowledge about architecture, geography, civil engineering, and public health—from those institutions was chosen due to their EH knowledge, taking into consideration their institutions' interest in EH and in having a dashboard to monitor EH, and due to their deep understanding of the EH-related databases and evidence. No prior experience with dashboards use was required. Four participants received a formal invitation via email with a description of the work. Due to the desire to capture the views of as many participants as possible, invited participants were given the opportunity to choose between a group interview and an individual interview. The members who consented to be interviewed had to communicate whether their co-workers would be part of the interview and provide their email addresses. All the participants received the instructions, the link for the interview platform, the interview template, and the informed consent before the interviews.

### Data Preparation and Visualization Formats

A search in local open-access databases—“Lisboa Aberta” (37), QualAR (38) and “Entidade Reguladora dos Serviços de Água e Resíduos” (39)—was performed to collect the available data for the 17 indicators validated as relevant to monitor EH in Lisbon (vide section Selected Indicators to Monitor EH in Urban Settings). The data were analyzed with Microsoft Power BI® software. A series of design cards were developed with different visualizations options (informed by relevant literature) for the group of indicators within each EH dimension (as defined in Figure 2). The design cards (see examples in the interview template available in Supplementary File) included visualization options such as bar plots and line graphs to represent time-series indicators with each data value or time reflecting the y-axis or x-axis (40). Tables were presented as options with more detailed information for all the indicators. Pie charts were used to visualize single series indicators to compare the behavior in each month during a year (41). And two-dimension maps were presented to display geographic information from databases.

Visualization is efficient if the large volume of data is perceived in a minimum amount of time (42). Following data visualization principles, the design cards included simple representations with no background, neutral colors like gray, dark red and green, no decorated frames, consistent layouts, and font size labels [by applying guidance from related literature (16, 41, 43)]. As Wästberg et al. (18) state, the use of form attributes such as shapes, or the use of a single color ranging from low to high intensity were used instead of rainbow color scales. Also, Yigitbasioglu and Velcu (44) argue that the excessive use of colors may confuse and distract the used, and cards were accordingly designed.

### Design of Semi-structured Interviews

Online semi-structured interviews were conducted using Microsoft Teams platform, between December 2020 and January 2021, and lasted for about 1 h. The screen was shared during the interview to allow the interviewees to add comments and interact with the interviewer and answer questions regarding the design cards. The interviewer wrote the answers in the template file. Due to institutional constraints of participants, the interviews were not recorded.

The semi-structured interviews consisted of four parts. In the first part, the interviewees had the chance to see four examples of public dashboards (45–48). Despite not being EH-related dashboards, these examples were chosen for being city or country dashboards used to monitor key urban metrics (10), and as a starting point for discussion. The interviewees were asked to select the preferred option and to indicate the characteristics in each example that they considered of key-value to the dashboard (see interview template available in Supplementary File).

In the second part, alternative design cards were presented for the group of indicators related to natural environment determinants (see Supplementary File). The visualizations options were created using Microsoft Power BI®, and they included simple representations to quickly highlight trends and deviations such as bar plots, line charts and tables (49). Every interviewee expressed his preference toward the most preferred option to visualize the indicators' data. Additionally, yes or no questions, and open-ended questions about the reasons for interviewees choices were used to understand which features and useful information should be added to each visualization for all the group of natural and built environment indicators.

In the third part of the interview, participants were invited to analyze the indicators in the built environment determinant, using the same questioning protocol adopted for the natural environment indicators (see Supplementary File). Nevertheless, the design cards in this case included different map-based visualizations created using Microsoft Power BI®, with the location of the metric measured by each indicator (50). Having different visualization options available in a group discussion allows the participants to refer to visualization elements to support their idea generation and discussion. The visualization options act as a conveyor of the data and as a reference point for all the participants in the group interview (51, 52). For the indicators without available data collected for Lisbon, the interviewees were asked to indicate which would be the most preferred way to visualize the needed data.

The last part of the interview was based on a set of yes/no and short open-ended questions to explore issues concerning the data quality, periodicity, and potential limitations. The interviewees were asked, for example, to state if the inclusion of the data source of each indicator or demographic data of Lisbon would be appreciated in the dashboard.

The answers provided by each participant were treated, analyzed, and aggregated to identify the requirements for an EH dashboard and emerging issues. Simple statistics, such as percentages, were calculated for the yes/no questions, and for the choice questions. For the short open-ended questions, a thematic content analysis was used to analyze the interviews. The analysis followed the principle of classifying and organizing data according to key and common concepts (53). Similar features identified by the interviewees were coded and organized to create a list of dashboard features with a set of requirements. The dashboard features and requirements are afterwards reported descriptively. To ensure the comprehensiveness of requirements, the working list was sent by email to each interviewee for validation. After receiving the feedback from the interviewees, the final list was adjusted considering their suggestions and re-sent for the interviewees for their knowledge.

## Results

### Elicitation of Users' Requirements

Three online group interviews were performed between December 2020 and January 2021 with members from Lisbon and Tagus Valley Regional Health Authority, from the Sustainability Cities Organization, and from the Lisbon City Council led to the involvement of 11 participants. Each interview lasted between 60 and 90 min. The interview with the Regional Health Administration included members from the sanitation and public health department (*N* = 3). From the Sustainability Cities Organization, members from the urban planning department participated (*N* = 3). The interview with the Lisbon City Council integrated members from the department of environment, energy, and climate change (*N* = 4) and from the department of environment and green spaces (*N* = 1).

The analysis of the interview' answers identified the preferences toward the options available in each part of the interview, shown in Table 1. The suggestions to visualize the indicators without collected data in Lisbon were also gathered in this analysis.

**Table 1:** User's preferences for each part of the semi-structured interview.

**Interview part**		**Users' preferences (%)**			
Part 1		**Option A** **Bristol dashboard**	**Option B** **Dublin dashboard**	**Option C** **Dublin dashboard**	**Option D** **Portugal dashboard**
		0	72.73	0	27.27
	**Indicators**	**Option A** **Line graph with monthly data**	**Option B** **Bar plot with monthly data**	**Option C** **Table with monthly data**	
Part 2	Air quality	45.45	54.55	0	
	Noise	36.36	45.45	18.18	
	“Água segura”	81.82^*^	9.09^*^	9.09^*^	
		**Option A** **Location with solid color filling all the area**		**Option B** **Location represented by a symbol with size proportional to the area**	
Part 3	Area of leisure parks and gardens	27.27	72.73		
	Area of community gardens	36.36	63.64		
	Energy poverty vulnerability index	9.09^*^	90.91^*^		
	Cycling roads	0	100		
	Number of road vehicles	18.18^*^	81.82^*^		
	Road network	0	100		

* *Preferences suggested for the indicators without collected data in Lisbon*.

Together with the users' preferences, a careful analysis for identifying, analyzing, and reporting themes within data in the answers to the open-ended questions of the interviews allowed to identify 11 requirements. The requirements were grouped within four groups of dashboard features to build a dashboard to monitor EH in Lisbon city. The summary of the requirements was sent by email to all the interviewees for validation. Six interviewees from the three organizations replied with some suggestions to improve the requirement description but stated an agreement with the proposed requirements. The suggestions were included in the list of requirements and the final list is presented in Table 2, with each group of requirements being explained afterwards.

**Table 2:** Requirements for a dashboard to monitor EH in the Lisbon city.

**Dashboard features**		**Requirement**	**Users' suggesting the requirement (%)**
Communicate data	1	Allow users to select which EH indicators are displayed.	100
	2	Use simple and easy to understand visualizations such as bar plots.	54.4
	3	Use discrete and distinct colors to orientate the reading of the data.	72.7
	4	Disaggregate data whenever possible.	100
Monitor performance	5	Include evidence-based standards, legal limits and political goals defined to improve EH.	100
	6	Provide selection of time periods, trends over time, and its changes.	100
Identify causes	7	Enable an interactive functional use of data.	81.8
	8	Enable users do “drill-down.”	70.0
	9	Provide maps with the zoom option.	100
Data quality	10	Include timely data measured with the same metrics.	100
	11	Provide sources of data.	72.0

### Communicate Data

In the first part of the interview the participants had the chance to analyse and compare four examples of city dashboards. The first identified requirement was the need to display the information in a way useful for the user. The dashboard should be designed to help the users to identify problems and to keep track of trends. These needs change according to the organization. Therefore, the dashboard must be customizable to enable the user to select which indicators to analyse and flexible enough to be used by different organizations (*Requirement 1*).

In the second part of the interview, interviewees were asked about their preferences regarding visualization options for natural environment indicators. All the interviewees agreed that the indicators from air quality should be visualized in the same graph, but no clear consensus was reached about the most preferred way to visualize them. Bar plots were the choice of 54.5% of the interviewees, while 45.4% preferred line graphs. A similar choice was observed for the indicators of noise dimension. Only for the water indicator was clear the agreement to visualize it using a line graph. The pie chart was considered a “hard to read” option, while tables were considered only as an additional feature to the initial graphic. Overall, the features more appreciated by the interviewees to visualize natural environment indicators were the use of easy-to-understand graphics, with adequate font size, and minimal information displayed. It would help the users to acknowledge at a glance an overall picture of all the indicators (*Requirement 2*).

In the third part of the interview, participants were asked about the built environment indicators. The data of these indicators were available in the public databases only in the map format. For the indicators without available data, a consensus was reached among the interviewees to use maps to visualize the data needed for the indicators. Design cards were presented to the interviewees including maps options with different types of legends and symbols to locate the area of the city under analysis. The maps in which colored circles identified the location with size proportional to the area were selected by 72.7% of the interviewees. The use of symbols with solid colors was a feature largely appreciated for the built environment indicators (*Requirement 3*).

While discussing the options for the indicators, the need to disaggregate the data was frequently discussed among the interviewees. Regardless of the option chosen, a graph or map, all interviewees acknowledged that it was essential to add the ability to access additional and detailed information of a particular indicator (*Requirement 4*). For instance, such feature would allow the user to see daily values in a specific location instead of monthly means for Lisbon city. The ability to depart from general data and overall assessments to more comprehensive and specific data would help understand the numbers' reasons.

### Monitor Performance

Two monitorization requirements were identified related to which information should be provided in a dashboard and how it should be presented. One of the features discussed was the added value of comparing the data being collected against evidence-based standards, legal limits, or political goals legal standards (*Requirement 5*). Users from the political and sustainability organizations were especially concerned with this aspect. The inclusion of a benchmark would help the end-users follow the indicators' performance, identify EH issues, and design strategies to deal with the situation.

The second feature to monitor the data is the ability to access historical data selecting specific periods for trends analyse (*Requirement 6*). This feature would be particularly important in the case of indicators in which they may expect to see fluctuations over seasons instead of daily or monthly alterations. Interviewees talked about wanting the possibility to choose a particular indicator and to select specific months and compare them with previous years.

### Identify Causes

Being able to identify causes or extreme events for any particular indicator was an element discussed in all the interviews. For 81.8% of the interviewees, the dashboard should be interactive and include features like the ability to see alerts whenever an outlier value was detected for a particular indicator selected by the user (*Requirement 7)*. Another feature discussed by more than 70% of the interviewees was the ability to “drill down” into the data or “drill up” to view general data (*Requirement 8*). In a drill-down dashboard, the users would be able to navigate different data layers to see specific and detailed information of a particular indicator without overcrowding the dashboard. Moreover, regarding the indicators visualized using maps, a consensus was achieved related to the inclusion of a zoom option (*Requirement 9*). The dashboard should be designed with the feature to view the data on specific areas by simply dragging the mouse over the part of the map the user wishes to explore. By focusing on the area of interest, users could easily detect potential problems or acquire a deeper understanding of trends.

### Quality Data

In the last part of the interview, participants discussed the quality of data to include in the dashboard, with key requirements being the availability of timely data and access to data: a lack of standardized data sources and of timely data with standard metrics were constraints faced by the organizations involved in this study (*Requirement 10*). All the interviewees stated that public databases' data are often perceived as out-of-date to be useful for short-term decision-making. Furthermore, data reported in distinct formats and using different metrics, together with interoperability issues between distinct data sources, lead to increased time spent analyzing the data and difficulted proper inference about the performance of an indicator. Seventy two percentage of the interviewees expressed the relevance of including the data source (*Requirement 11*). Having access to the original database (i.e., the source of data) would increase the users' trust as it would enable users to check inconsistent values or clarify potential doubts.

## Discussion

Organizations are slowly adopting health and urban dashboards as tools to support better decision-making and deliver large-scale feedbacks to healthcare providers and policymakers (54). Such decision aiding tools are often designed without suitable evidence and structured procedures (55). To address this gap, this study adopted an approach informed by scientific evidence and expert opinions, within a user-centered approach, to identify a set of requirements to inform the design of a dashboard to monitor EH in the Lisbon city.

Our findings provide a first step toward the design of a user-friendly tool with the potential to inform EH interventions. Results from this study highlight recurring data constraints related with built and natural environment indicators, with such constraints impacting the design of a dashboard for the Lisbon context.

As Tao et al. (35) and Mohammadi et al. (56) suggested, the selection of the information to be displayed in a dashboard should be performed through social interaction. Such selection process creates opportunities for experts and policymakers to lend their views and expertise and indirectly increase their trust in the tool. The set of indicators used within the user-centered approach adopted in this study was selected through a literature review and through experts' participation reported in two previous studies. Although the interviewed participants of this study were initially cautious about the inclusion of such heterogeneous data into a single dashboard, all indicators were deemed relevant, and no additional indicators were suggested. Furthermore, the requirements derived from this study are based on indicators selected to monitor EH. Still, they can serve as a guideline for other contexts trying to build tools using indicators with similar metrics or features. We discuss results from the lens of methods, from the EH lens, and discuss the study's strengths and limitations.

### Elicitation of User's Requirements

A successful design of an efficient dashboard demands that user requirements are fulfilled (57, 58). Therefore, these requirements must be clearly identified and easily understood. Our findings suggest that combining group interviews with the use of design cards is a suitable approach to engage end-users in the early stages of dashboard design.

Consistent with prior research (32, 59), the user-centered approach allowed the participants to understand the dashboard's aim and easily define the elements and requirements for its design. The participants drew on their professional expertise to critically analyse the data, and were able to collaboratively reach an agreement about the most preferred visualization option for all the indicators and about the critical features to be included in the dashboard.

The design cards approach proved to be useful in helping potential users to transmit their ideas on how to communicate the data in a tangible form. Having different options to compare, the interviewees were able to easily identify which data was missing or needed to be updated, and at the same time, define features for each indicator. In the case of indicators without available data for Lisbon, the interviewees were comfortable in discussing about what they expected to see, about which data should be collected, and about the best way to visualize it. For instance, for the indicator “number of cars,” a consensus was reached to use a map to visualize the indicators and collected data disaggregated by type of fuel and type of public or private vehicle.

Overall, the interviewees sought that a combination of simple and interactive visualizations with the ability to access detailed visualizations would enable a deeper analysis of indicators' data. The end-users' preferences were aligned with the data visualization research, which has shown that users can quickly understand data displayed in both bar plots and line graphs (16, 60, 61). For example, bar plots or line graphs should be used to visualize the overall trends of the indicators, and also to allow an access to tables with detailed data for specific locations and periods.

A shared agreement was achieved about the requirements to be respected by a dashboard that can help identifying patterns, trends and correlations between indicators and making sense of data. Features such as zooming options and inclusion of legal limits or local goals were recalled by all participants. Having a benchmark would help the user to get a clear notion of how the indicator is evolving and support anticipating different scenarios and strategies to deal with it (62). Another critical element discussed was data quality. To inform policies and to determine whether a policy is being effective, different data sources need to be combined. Investing in identifying databases for all the indicators, requesting access to the databases, and standardizing metrics is essential to build a dashboard (14). The lack of standardization hampers a potential comparison of datasets from different sources and may lead to unreliable data. The comparability of periodical and geographical data is highly important, as benchmarking could be required for decision-making (9).

Furthermore, it is important to note that an essential requirement for a successful dashboard is to provide dynamic information. Also, a dashboard should enable users to tailor the indicators' information in which they are most interested. The fulfillment of this requirement suggests the design of a dashboard flexible enough to be implemented in different organizations.

Microsoft Power BI proved to be a suitable software to support this integrated approach. Power BI has a library with several standard visualization options to inform the development of the design cards and the dashboard's design. This software had the advantage of connecting a large volume of data and model it in different ways (63). It also revealed great compatibility with the different databases used in this study.

Finally, as Saarijarvi and Bratt (64) show in their study, online interviews are a suitable alternative method to face-to-face interviews. The online group interviews implemented in this study have proved to be successful for the purpose of attaining the objectives set in this study.

### Environmental Health Implications

This work provides key information for decision-makers creating a new system to monitor EH in the Lisbon urban area. Although available urban dashboards include some of the environmental indicators deemed key to monitoring EH, they focus on urban management instead of health monitorization. For this reason, the engagement of experts as potential end-users in an integrated approach to design a dashboard reflects its unique ability to help stakeholders and decision-makers focusing on both health needs and environmental drivers (65, 66). Engaging end-users from organizations with different perspectives and roles regarding monitorization of EH in Lisbon allowed us to gather the different needs and to identify the issues about data collection. Requirements such as the inclusion of legal limits for benchmarking or the need for up-to-date databases with similar metrics showed the concern with the comparability of data to support environmental decisions. The data should be presented to the user in a way to allow a detailed search to compare and summarize the information. A clear understanding of the relationships between different environmental indicators would help identify locations, detect potential sources, define the trends, and serve as a baseline for further investigation to inform EH interventions.

The implementation of a dashboard to monitor EH at the local level can be a solution to catalyze efforts to improve EH at the national level and can be replicated in other settings. Moreover, dashboards at lower geographic level can help to understand the impact of local policies on health, and it can pave the way for cross-sectional efforts between researchers, industries, and policymakers to improve (local) EH interventions (10).

### Strengths and Limitations

The methodology implemented in this study was informed by existing related research (10, 11, 67, 68) using user-centered approaches to facilitate mutual learning and shared understanding among potential end-users. By involving different end-users in the early-stage design, the requirements provide insight into how a dashboard to monitor EH can be developed in practice, promote real-world usability of new dashboards, as well as allow for proactively dealing with user experience issues; and potential users may be more willing to use the dashboard and participate in future dashboard construction stages. To the best of our knowledge, this study is the first attempt to describe an approach to inform the design a dashboard to monitor EH. Although urban dashboards are increasingly becoming a tool for policymaking, no urban EH dashboard was found as a reference. The users involved in this study revealed a shared awareness of the need for a dashboard to monitor EH and agreed with the indicators identified as relevant to monitor EH in Lisbon.

Despite these contributions, several important lessons were learned throughout this study. First, the pandemic required the authors to adapt the methods and adopt online and interactive interviews instead of face-to-face interviews. A series of design cards were prepared for friendly visualization, helping the participants to engage in the interview in such a remote setting. Another challenge that is common when implementing new tools in healthcare and in political organizations is the difficulty felt by users when envisioning the design of new tools that they never conceived before. For instance, designing an EH dashboard for the Lisbon city can be challenging for some users when asked to foresee how they wanted to view and use data. The use of design cards with visualization options was effective to overcome the difficulty of envisioning visualization format but could introduce bias since users often asked for a mix of data or agreed that all options would work well.

Finally, some questions can be raised regarding the generalization of findings to other contexts or dashboards because of the small sample of interviewees. This study involved a non-probabilistic sample from organizations developing EH interventions and regulations in the Lisbon city. Such kind of sample may result in biases since those are the most concerned with implementing tools that help improve EH in Lisbon city and may be more prone to make time to participate. Although an agreed set of requirements was identified, the number of interviewees was too small to enable statistical analysis of the answers of the three groups of interviewees. Nevertheless, it was successful in obtaining from EH experts the requirements most relevant for a dashboard to be used by their organizations. The discussion format provided valuable insights about the dashboard's formats and contents and about EH monitoring in urban settings.

In order to further understand if the requirements identified in this study are what is required for the dashboard to work, it would be valuable to build the dashboard using PowerBI®, implement it together with the involved organizations, and to further interact with the participants so as to adjust and validate the dashboard.

## Conclusions

Making available EH dashboards to environmental, healthcare, sustainable, and policy organizations has the potential to promote evidence-based policymaking and to contribute to the improvement of quality of life or health outcomes. It is undeniable that shortcomings in (dashboard) requirements elicitations can lead to inadequate implementations (69). The inclusion and collaboration of potential users are essential in the phase of requirements' elicitation. Throughout interactive and targeted approaches, after selecting the indicators to be included in the dashboard, the authors identified the requirements to design a urban EH dashboard with the potential to help organizations and policymakers planning successful interventions. In this study, an integrated approach was specifically followed to engage potential users from diverse organizations in the collaborative development of design requirements for a dashboard to monitor EH in Lisbon city. Critical insights were obtained from data needs, visualization preferences and other discussions with potential end-users, including about how to communicate data, monitor indicators' performance, and identify causes and issues of data quality.

This study can be looked upon as a step within a participatory framework to build EH dashboards. The integrated approach described can contribute to the development of easily comprehensible visualizations of environmental data for urban settings, based on user and organizations' needs; and it can guide the design of health and/or urban dashboards and serve as a reference for other researchers to design new tools.

## Data Availability Statement

The raw data supporting the conclusions of this article will be made available by the authors, without undue reservation.

## Author Contributions

MS and MO: conceptualization and writing—original draft preparation. MS: investigation and visualization. PN and MO: validation. MO: writing—review and editing. AT, MO, and PN: supervision. All authors have read and agreed to the published version of the manuscript.

## Funding

MS was supported by the Portuguese Foundation for Science and Technology (FCT) and Valorsul S.A. under the scholarship number PDE/BDE/120465/2016. MO was supported by the Portuguese Foundation for Science and Technology under the MEDI-VALUE project (Grant No. PTDC/EGE-OGE/29699/2017). MS and MO acknowledge support from the Center for Management Studies of Instituto Superior Técnico, a research center funded by FCT project UIDB/00097/2020. MS and PN acknowledge support from ISAMB, a research center funded by FCT project UIDB/04295/2020.

## Conflict of Interest

The authors declare that the research was conducted in the absence of any commercial or financial relationships that could be construed as a potential conflict of interest.

## Publisher's Note

All claims expressed in this article are solely those of the authors and do not necessarily represent those of their affiliated organizations, or those of the publisher, the editors and the reviewers. Any product that may be evaluated in this article, or claim that may be made by its manufacturer, is not guaranteed or endorsed by the publisher.
