# Supplementary material for: Setting Requirements for a Dashboard to Inform Portuguese Decision-Makers About Environment Health in an Urban Setting
Source: Front Public Health. 2022 Jun 10;10:837433. doi: 10.3389/fpubh.2022.837433 (PMC9226409; doi:10.3389/fpubh.2022.837433)
Supplement: Supplementary file 1 [file Data_Sheet_1.PDF]

Interview template

PART 1- ANALYSIS OF EXISTING URBAN AND HEALTH DASHBOARDS

In the first part of the interview, the interviewees are asked to analyse four examples of public dashboards being used to monitor urban metrics. The screens used in the interview are the original ones, in English or in Portuguese.

1.1 The following design cards contain examples of public dashboards. Please select the option that you most prefer.

Option A	☐
Option B	☐
Option C	☐
Option D	☐

Option A

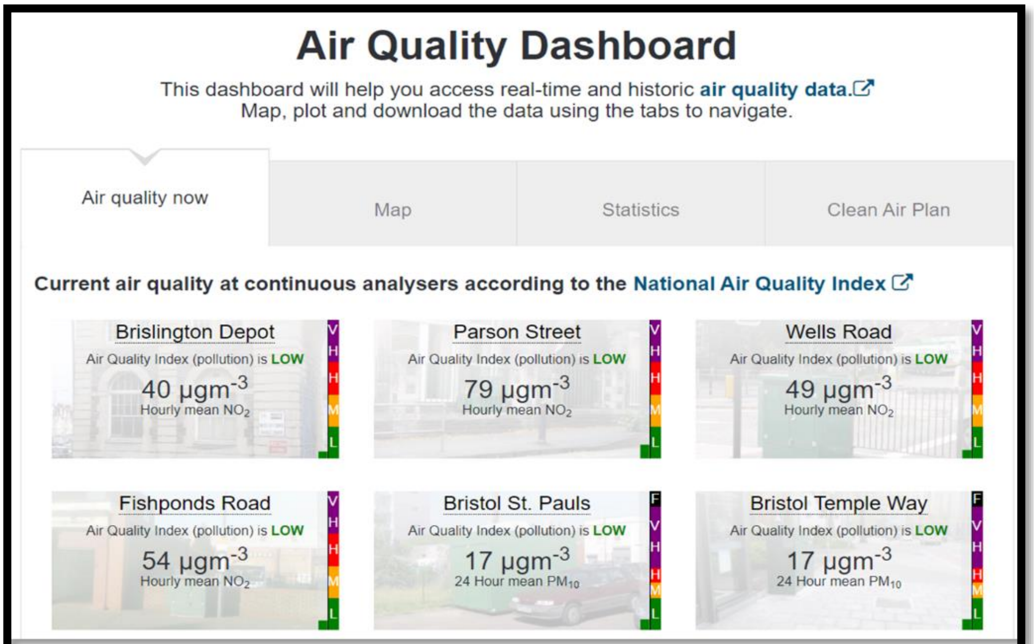

https://opendata.bristol.gov.uk/pages/air-quality-dashboard-new

Option B

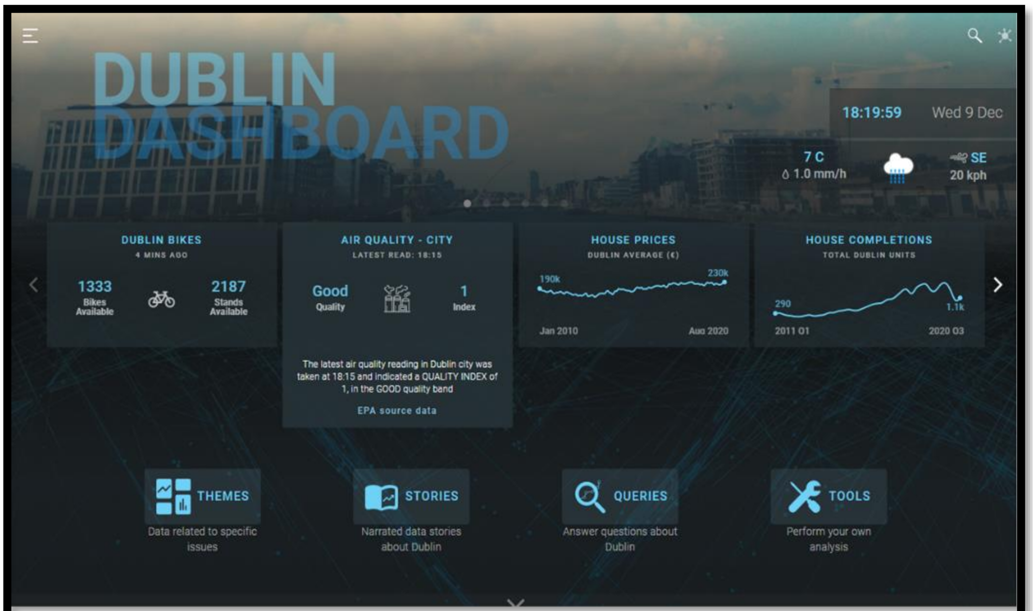

https://www.dublindashboard.ie/

Option C

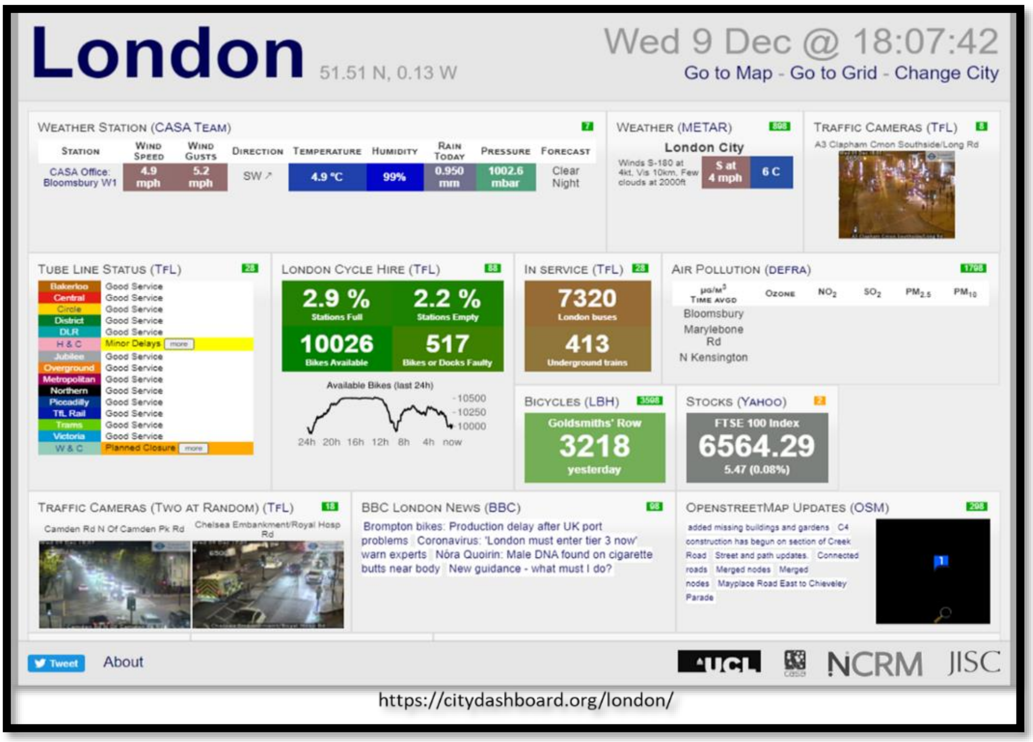

https://citydashboard.org/london/

Option D

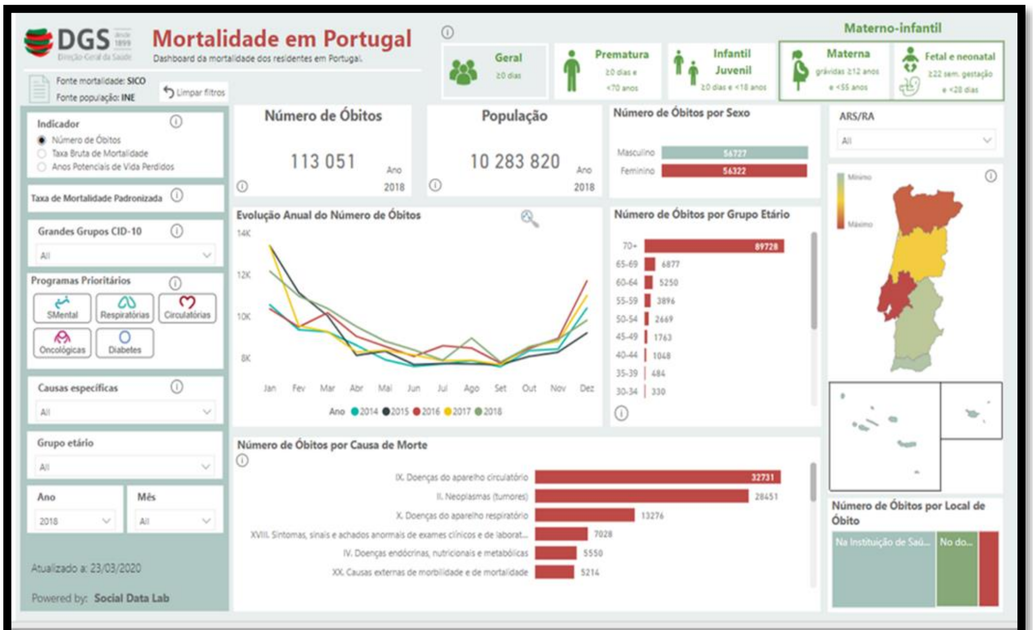

https://www.sns.gov.pt/noticias/2019/11/11/dgs-plataforma-da-mortalidade/

1.2 Can you please justify your choice and state the feature(s) that you appreciate more?

1.3 Is there any feature from the other examples that you particularly appreciate?

PART 2- ANALYSIS OF THE NATURAL ENVIRONMENT INDICATORS

In this part of the interview a design card approach is used. Different visualization options created using Microsoft Power BI® of graphs are presented, and interviewees are asked to choose the most preferred representation for the natural environment indicators.

Taking the air quality indicators as the example for the discussion, interviewees are asked to answer the following questions. The screens presented are the original ones used in the interview, in Portuguese.

2.1 Do you prefer to visualize all the indicators at the same time or separately?

2.1.1. In the case you prefer to see all the indicators at the same time, the following design cards depict all the indicators from air quality. Can you please select the most preferred option to visualize the indicators?

Option A	☐
Option B	☐
Option C	☐

<u>Option A</u>- Line graph with monthly data.

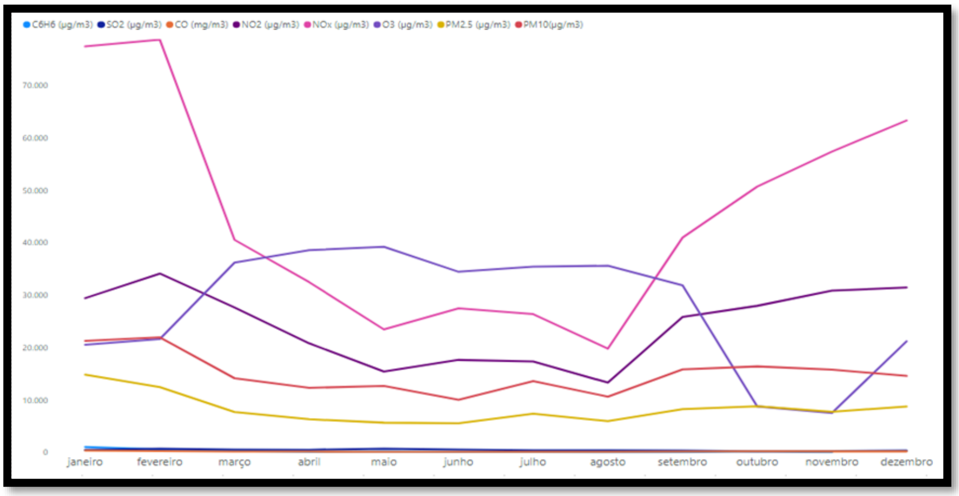

Option B- Bar plot with monthly data.

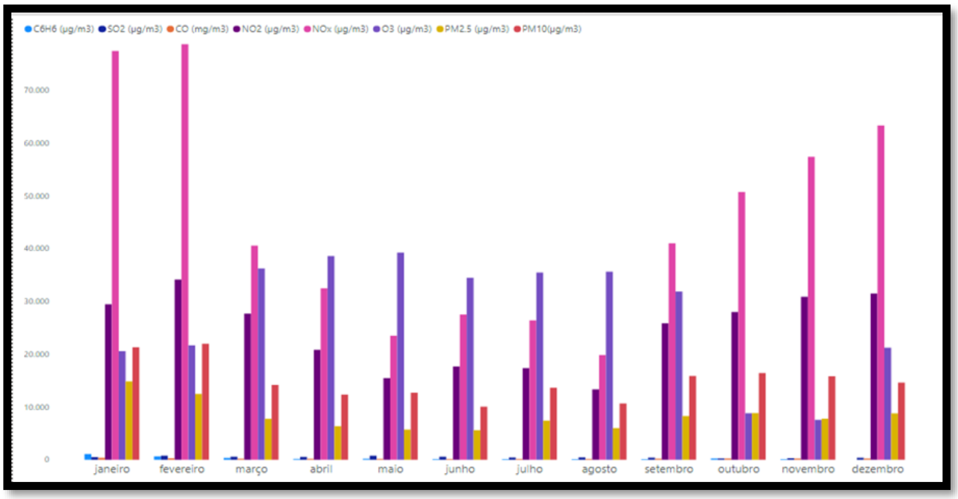

Option C- Table with monthly data.

Mês	C6H6 (µg/m3)	SO2 (µg/m3)	CO (mg/m3)	NO2 (µg/m3)	NOx (µg/m3)	O3 (µg/m3)	PM2.5 (µg/m3)	PM10(µg/m3)
janeiro	1.088,80	507,46	379,80	29.475,44	77.510,75	20593	14.861,25	21.323,16
fevereiro	632,20	771,99	288,39	34.173,73	78.792,41	21686	12.483,28	21.995,40
março	354,00	573,16	208,38	27.706,99	40.601,80	36267	7.761,97	14.190,53
abril	161,20	540,98	193,46	20.848,53	32.516,02	38622	6.361,43	12.352,54
maio	196,00	764,53	177,95	15.481,86	23.517,73	39285	5.716,74	12.718,38
junho	137,30	572,06	151,92	17.690,30	27.541,84	34508	5.598,53	10.067,38
julho	131,00	425,79	159,71	17.377,73	26.419,74	35498	7.411,44	13.662,69
agosto	23,00	446,37	158,53	13.352,57	19.844,36	35662	6.009,42	10.669,95
setembro	76,30	402,24	206,89	25.869,80	41.033,85	31900	8.282,54	15.892,51
outubro	243,20	252,70	241,91	28.012,59	50.770,87	8820	8.843,35	16.447,15
novembro	19,50	300,70	234,18	30.905,28	57.443,66	7562	7.785,60	15.833,96
dezembro		381,26	234,17	31.517,01	63.381,11	21243	8.797,10	14.633,73

2.1.2. In the case you prefer to see the indicators separately, the following design cards depict the case for only one indicator from air quality. Could you please select the most preferred option to visualize the indicator?

Option A	☐
Option B	☐
Option C	☐
Option D	☐

Option A- Line graph with monthly data.

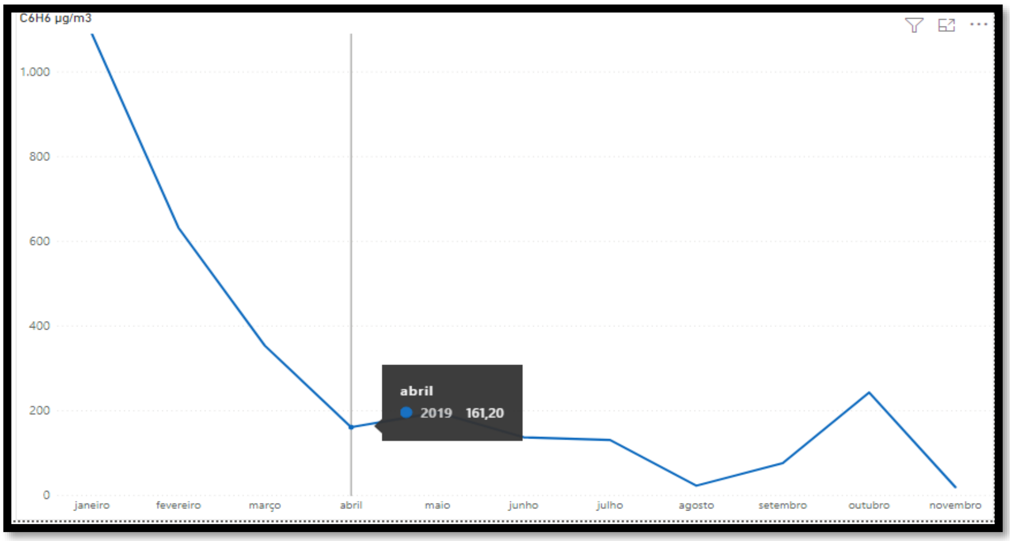

Option B- Bar plot with monthly data.

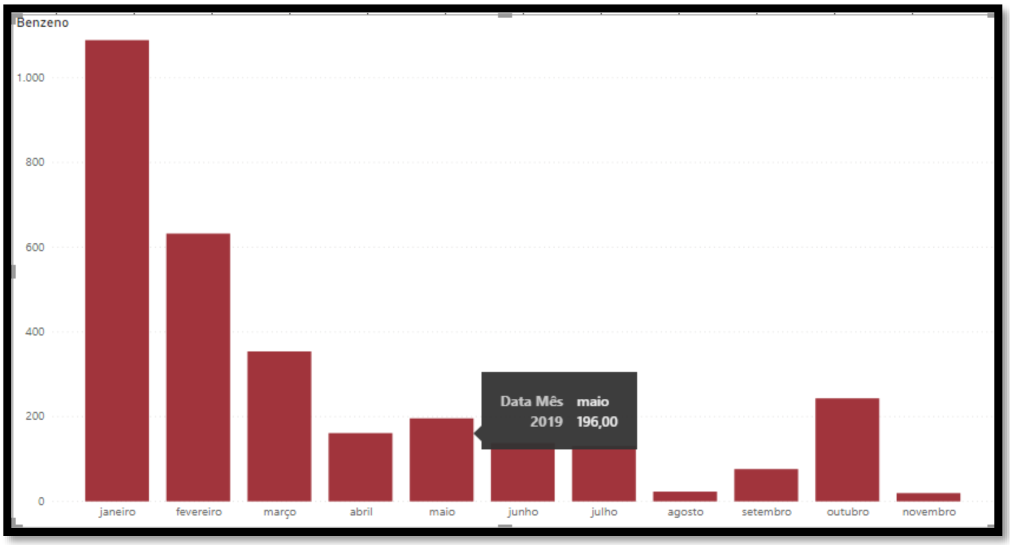

<u>Option C</u>- Pie chart with monthly data.

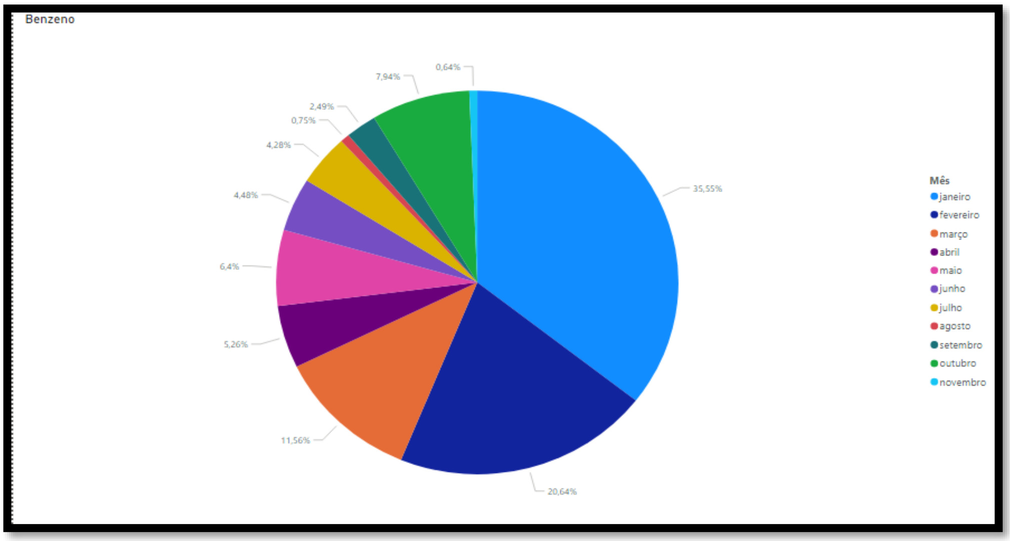

<u>Option D</u>- Table with monthly data

Mês	2019
janeiro	1.088,80
fevereiro	632,20
março	354,00
abril	161,20
maio	196,00
junho	137,30
julho	131,00
agosto	23,00
setembro	76,30
outubro	243,20
novembro	19,50

2.2 Can you please justify your choice?

2.3 With which periodicity would you like to visualize the data (for instance, daily, monthly, quarterly)?

2.4 Do you consider it useful to include benchmark information? Do you have any further comments?

2.5 The noise indicators are available in a map-format. Do you consider this visualization option the most preferred option for these indicators?

2.5.1 Would you appreciate more to visualize these indicators in a graph format? In that case, in which type of graph?

2.6 The indicator "Água Segura" is represented by an annual value. Which would be the best way to visualize this indicator?

PART 3- ANALYSIS OF THE BUILT ENVIRONMENT INDICATORS

This part of the interview also makes use of a design card approach. Different visualization options created using Microsoft Power BI® of graphs are presented, and interviewees are asked to choose the most preferred representation for the indicators from the built environment determinant. The screens used in the interview are the original ones, in Portuguese.

Taking the "Area of green spaces" and "Cycling roads" indicators as the examples for the discussion, please answer to the following questions (the same logic).

3.1. Could you please select the most preferred option to visualize the indicator "Area of green spaces"?

Option A	☐
Option B	☐

<u>Option A</u>- Location with solid color filling all the area.

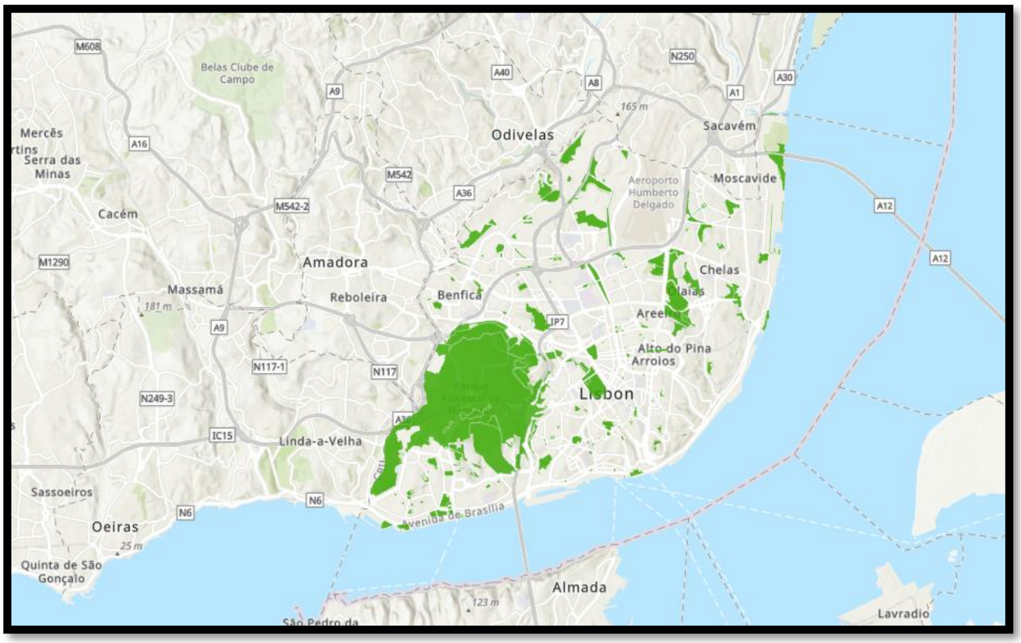

<u>Option B</u>- Location represented by a symbol with size proportional to the area.

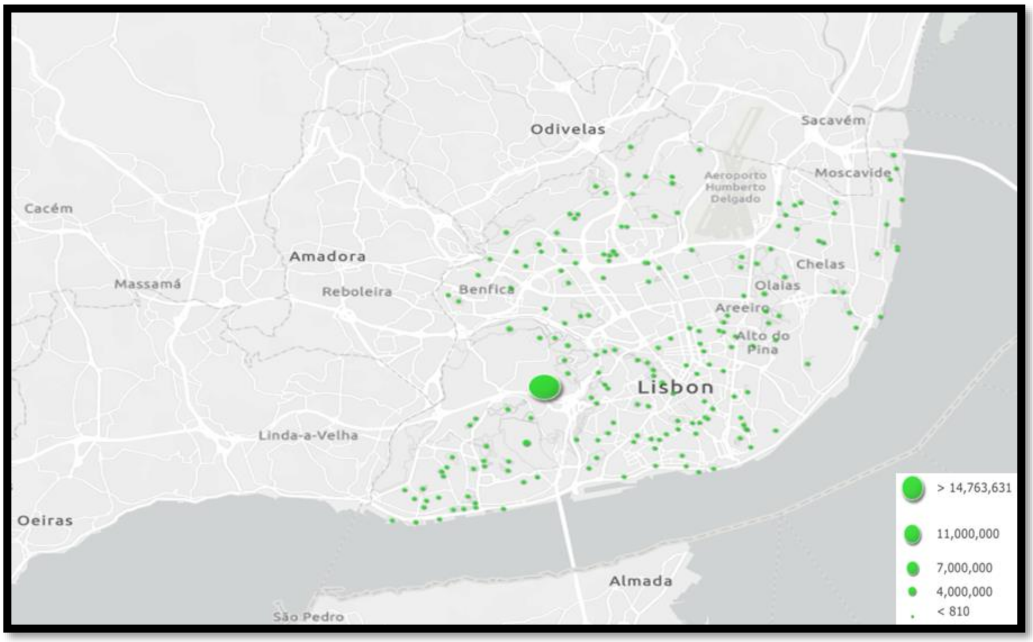

3.2. Could you please select the most preferred option to visualize the indicator "Cycling roads"?

Option A	☐
Option B	☐

<u>Option A</u>- Location with line with solid color.

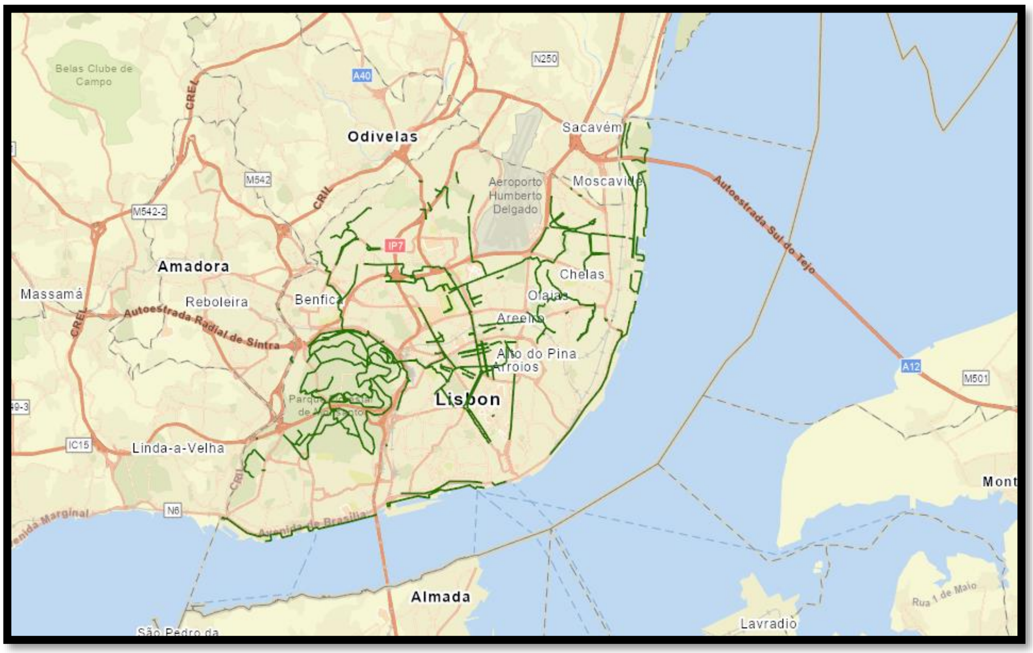

Option B- Location represented by lines with different colors for each type of cycling road.

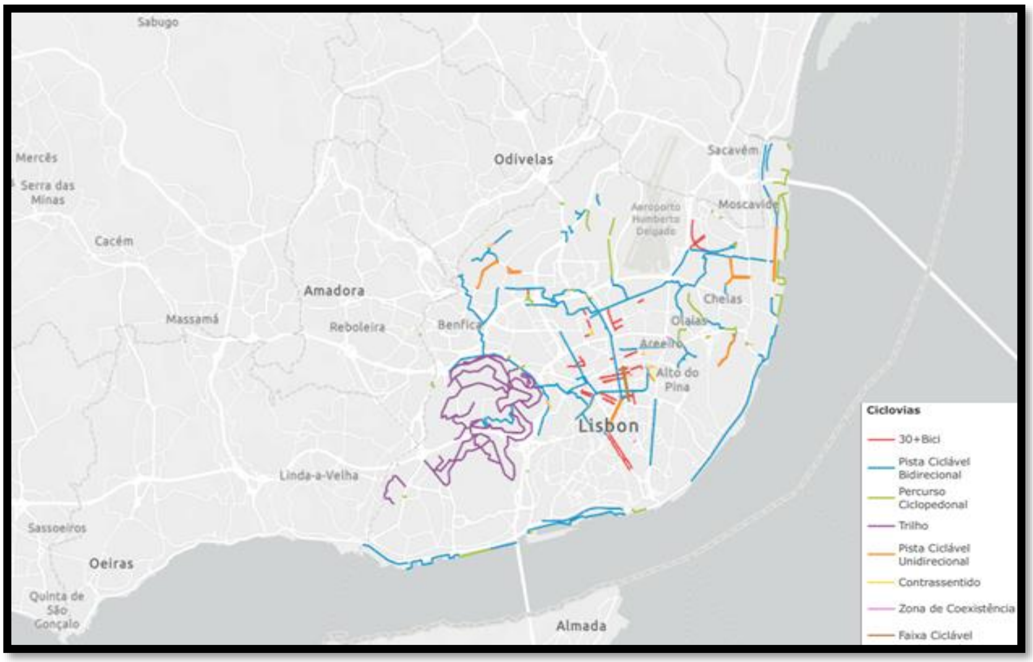

3.3 Can you please justify your choice?

3.4 Do you consider important to include a zoom option?

3.5 Which type of information do you consider more important to be accessible in this visualization?

3.6 Do you have any further comments?

3.7 Having data collected for the "Energy poverty" and "Number of road vehicles" indicators, which type of visualization option would be suitable to visualize these indicators?

PART 4- ANALYSIS OF GENERAL FEATURES OF THE DASHBOARD

This part of the interview is based on a set of yes/no and short open-ended questions to explore issues concerning the data quality, periodicity, and potential limitations.

4.1 Should the data source be included for each indicator?

4.2 In the absence of legal limits and targets for the indicators, what kind of information do you think should be included? For example, percentage of increase/decrease of cycling roads over the years.

4.3 Do you consider it useful to show the periodicity of data collection? Where would you like to see that information?

4.4 Should the dashboard include historical data or only data from a defined period of time?

4.5 **Is there any demographic information that you consider important to include?** For example, population number or area of city.

4.6 Is there any kind of information that should NOT be included in the dashboard?
